# Antibiotics as Adjunctive Therapy in the Non-Surgical Treatment of Peri-Implantitis: A Systematic Review and Meta-Analysis

**DOI:** 10.3390/antibiotics11121766

**Published:** 2022-12-07

**Authors:** Maria Gabriella Grusovin, Alberto Pispero, Massimo Del Fabbro, Matteo Sangiorgi, Massimo Simion, Martina Stefanini, Elena Maria Varoni

**Affiliations:** 1 Dipartimento Odontoiatria, Università Vita Salute “S. Raffaele”, 20132 Milan, Italy; 2 Libera Professionista in Gorizia (Dental Private Practice), 34170 Gorizia, Italy; 3 Dipartimento di Scienze Biomediche, Chirurgiche ed Odontoiatriche, Università Degli Studi di Milano, 20122 Milan, Italy; 4 IRCCS Fondazione Ca’Granda Ospedale Maggiore Policlinico, 20122 Milano, Italy; 5 Dipartimento di Scienze Biomediche e Neuromotorie, Università di Bologna,40123 Bologna, Italy

**Keywords:** peri-implantitis, systematic review, therapy, antibiotics, peri-implant diseases, clinical studies

## Abstract

The role of antibiotics as adjunctive therapy in the non-surgical treatment of peri-implantitis is uncertain. The aim of this systematic review of randomized controlled trials was to assess the efficacy of antibiotic therapy, local or systemic, as an adjunctive to the non-surgical therapy of peri-implantitis. Primary outcomes were: implant success rate and complications, changes in radiographic bone level, probing pocket depth (PPD), probing attachment level (PAL), bleeding on probing (BOP) and peri-implantitis resolution. Six studies were included: two using topical and four systemic antibiotics. Adjunctive local antibiotics improved PPD (mean difference (MD) = 0.6 mm; 95% CI 0.42–0.78), BOP (MD = 0.15% (95% CI 0.10, 0.19)) and the success rate (risk ratio = 9.89; 95% CI 2.39–40.84). No significant difference in bone level and success rate were found with the use of systemic antibiotics, although they appeared to improve PPD (MD = 1.15 mm; 95% CI 0.31–1.99) and PAL (MD = 1.10 mm; 95% CI 0.13–2.08). Within the limitations of this review, the adjunctive local antibiotics showed improved outcomes in terms of success rate, PPD and BOP, while adjunctive systemic antibiotics improved PPD and PAL only. Peri-implantitis resolution was about 20–30% using adjunctive local antibiotics, whilst it ranged from 2% to 65% with adjunctive systemic antibiotics. Findings are still controversial, since they are based on few studies with high heterogeneity, at the uncertain or high risk of bias and involve few patients. Non-surgical debridement and maintenance periodontal support therapy remain pivotal and the adjunctive use of antibiotics for peri-implantitis cannot be routinely recommended, even considering the increasing concern on antibiotic resistance.

## 1. Introduction

The term “peri-implantitis” was introduced in the 1960s for describing infectious pathological conditions of the peri-implant tissues, and in the 1990s during the 1st European Workshop on Periodontology, it was further specified how the term describes an inflammatory process that leads to tissue destruction around osseointegrated implants with the formation of a peri-implant pocket and bone loss. More recently, the 2017 World Workshop on Periodontal Diseases defined peri-implantitis as “a plaque-associated pathological condition occurring in tissues around dental implants, characterized by inflammation in the peri-implant mucosa and subsequent progressive loss of supporting bone” [1]. While peri-implant mucositis is considered to precede peri-implantitis, the patient-related and the site-related conditions leading to the progression from mucositis to peri-implantitis are still unknown. Peri-implantitis may occur early after implant placement and it appears to progress in a non-linear pattern [1]. The biofilm represents the main etiological factor. Poor plaque control and not attending regular maintenance therapy are among the key risk factors/indicators for the development of the disease [2]. Anti-infective therapies showed to be effective in decreasing inflammation around implants and reducing disease progression [1]. Different types of non-surgical and surgical treatments are described in the literature, but little evidence is available to support a gold standard therapy [3].

Mombelli and Lang (1992) [4] firstly proposed the use of antibiotics after the mechanical disruption of the biofilm to facilitate eradication of bacteria from implant surfaces and from peri-implant soft tissues. In 1997, Lang and co-workers recommended a maintenance care system based on the severity of peri-implant disease; the Cumulative Interceptive Supportive Therapy (CIST) was a protocol based on scientific literature and clinical experience, although no randomized controlled trials were available [5]. Mechanical cleaning was considered the first step, and then antiseptics, antibiotics and surgical intervention were suggested as additional treatments according to the deepening of the pockets and the progression of bone loss. Mechanical therapy was suggested as the treatment only for pockets with a periodontal probing depth (PPD) of less than 4 mm, while pockets deeper than 5 mm and with bone loss were treated also with ornidazole and metronidazole as systemic antibiotics and tetracycline fibers as topical antibiotic therapy [5].

The appropriate use of antibiotics is considered a worldwide priority [6] due to the rise in antimicrobial resistance [7], and the use of antibiotics in non-surgical treatment of peri-implantitis remains, to date, controversial. While some case reports showed additional benefits of antibiotics administered both locally [8] and systemically [4,9,10,11], the efficacy of the adjunctive use of antibiotics is currently not clear, as suggested by two previous systematic reviews [12,13]. On the other hand, some potential benefits were found in two further systematic reviews that addressed the adjunctive role of antibiotics in both non-surgical and surgical therapy of peri-implantitis [14,15]. However, few studies with high risk of bias were included; in particular, not only randomized studies were considered, therefore well-designed clinical trials were highly recommended to sustain the efficacy of antibiotics. Furthermore, the reviews on peri-implantitis treatment previously published suffer from a lack of consistency in disease definition within the analyzed studies. Disease definition and related correct diagnosis represent the cornerstones to proceed with the appropriate treatment and to evaluate its success. So far, no review has considered only randomized controlled clinical trials (RCTs) applying the most recent definition of peri-implantitis, as proposed by the 2017 World Workshop on Periodontal Diseases [1]; moreover, new randomized studies have been published on the topic in last few years.

The aim of this systematic review was to evaluate the efficacy of the adjunctive use of antibiotics, both local and/or systemic ones, in the non-surgical treatment of peri-implantitis, as defined by the 2017 World Workshop on Periodontal Diseases.

## 2. Results

**Figure 1:**
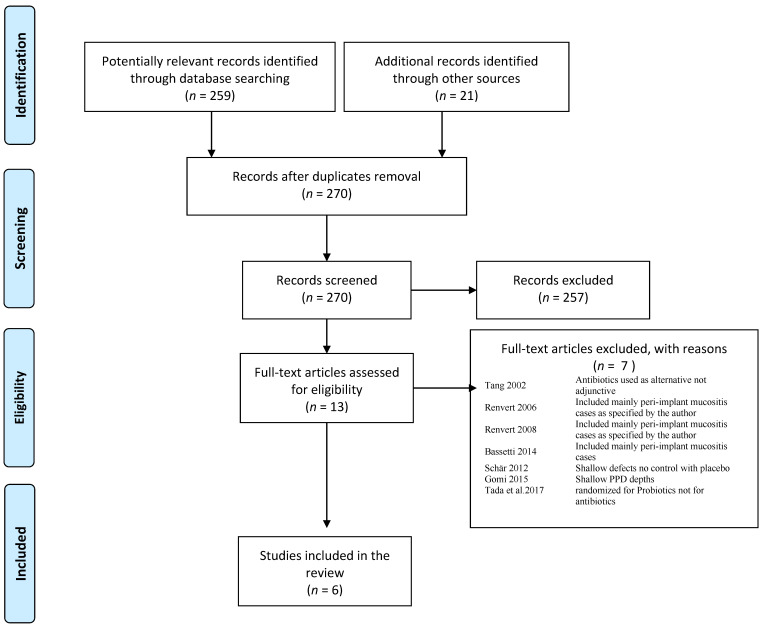
Flow diagram of the screening process following PRISMA [18,19,20,21,22,23,24].

The flowchart of the screening process is shown in Figure 1. Six studies were included in the systematic review. One study [16] investigated two different local antibiotic therapies and these were analyzed separately (indicated in the data analysis as “Park 2021a” and “Park 2021b”). Overall, 391 patients (375 implants) were included, excluding the clinical trial of Blanco et al. 2022 [17], in which the total number of implants was not clearly stated.

A description of the studies is summarized in Table 1 and Table 2.

Smoking habits were described in all studies and two of them included only no smokers [25] or light smokers [16]. History of periodontal disease was recorded in three studies [17,26,27] and the groups were comparable at baseline. Implant type was not described in two studies [17,25]. One study reported the use of implants with rough surfaces [28]; other types of implants were included in the other three studies, where implant brand, but not the implant surface, was specified [16,26,27].

Non-surgical treatment followed different protocols for debridement and metallic instruments, including stainless steel curettes, which were used in two studies [17,26], while dedicated ones, such as Teflon curettes, were used in the other three [25,27,28] and one study did not report the type of instrumentation employed [16]. In two studies, chlorhexidine was additionally applied during the treatment [17,28] and, in two other studies, during the maintenance phase [26,27]. Air-polishing was employed in one study [26]. Maintenance protocols were described in all studies, although differing among each other. The use of local anesthesia was reported in three studies [16,25,26].

**Table 1:** Description of study design and intervention for the included studies.

Publication	Design/Follow-Up (months)/Population	Administration	Case Definition	Pretreatment	Treatment
Butcher 2004 [28]	Parallel RCT/4 months/Not available	Local Doxycycline 8.5%	Peri-implantitis with bone loss exceeding 50% on X-rays, PPD > 5mm	Full mouth debridement + subgingival irrigation 0.2% Chlorhexidine 2 to 18 weeks before baseline	Irrigation with chlorhexidine digluconate 0.2% solution hand plastic instrument +/− 8.5% doxycycline hyclate (Atridox, Block Drug Corporation, Inc, Jersey City, NJ, USA) (test)
Park 2021a [16]	Parallel RCT/3 moths/Republic of Korea	Local metronidazole benzoate (201.0 mg)-minocycline hydrochloride (10 mg)	One Implant per patient placed at least 1 year previously, with PPD ≥ 5 mm, BoP (bleeding on probing), SoP (suppuration on probing), and the presence of peri-implant bone loss on X-rays	No pretreatment	Non-surgical debridement was carried out using an ultrasonic scaler and oral hygiene products. Local delivery of antibiotics, in the sulcus, was performed at baseline, 1, 2, and 3 weeks after baseline, while no further treatment was applied in the nonsurgical treatment group (debridement only)
Park 2021b [16]	Parallel RCT/3 moths/Republic of Korea	Local minocycline hydrochloride (10 mg)	One Implant per patient placed at least 1 year previously, with PPD ≥ 5 mm, BoP, SoP, and the presence of peri-implant bone loss on X-rays	No pretreatment	Non-surgical debridement was carried out using an ultrasonic scaler and oral hygiene products. Local delivery of antibiotics, in the sulcus, was performed at baseline, 1, 2, and 3 weeks after baseline, while no further treatment was applied in the nonsurgical treatment group (debridement only)
Blanco 2021 [17]	Parallel RCT/12 months/Portugal	Systemic metronidazole 500 mg 3 times a day for 7 days	Bop and/or SoP, PPD ≥6 mm and ≥3 mm of peri-implant bone loss on X-rays	Supra gingival debridement, elimination retentive factors. FMPS ≤ 20%	Local anesthesia supra- and submucosal stainless-steel U.S. + chlorhexidine 0.12% irrigation + stainless steel curettes to remove granulation tissue//in the test group 500mg metronidazole 3 times day for 7 days
De Waal 2021 [26]	Parallel RCT/3 moths/Netherlands	Systemic metronidazole 500 mg + amoxicillin 500 mg 3 times a day for 7 days	BoP, SoP and PPD ≥5 mm and ≥2 mm of peri-implant bone loss on X-rays	Full mouth debridement in one to five sessions. One mouth rinse with 0.12% CHX + 0.05% cetylpyridinium chloride before each session	Implants were supra- and submucosally cleaned using an air polisher with sub-gingival tip, i.e., EMS Air-flow^®^ with erythritol-based powder containing chlorhexidine (Air-Flow ^®^ Powder PLUS, EMS) and ultrasonic instruments (PL1 and PL2 instruments, EMS Piezon^®^; only on exposed screw threads, never on smooth implant surfaces). In one to five sessions. In the test group systemic, amoxicillin and metronidazole (500/500 mg, 3 times daily for 7 days)
Shibli [25]	Parallel RCT/12 months/Brazil	Systemic metronidazole 400mg + amoxicillin 500mg 3 times a day for 14 days	PPD > 5mm; bone loss > 4mm and BoP/suppuration; <50% bone loss	Prophylaxis/oral hygiene instruction	Teflon curettes single appointment//in test group metronidazole, (400 mg) and amoxicillin (500 mg) three times a day for 14 days
Polymeri 2022 [27]	Parallel RCT/3 moths/Netherlands	Systemic amoxicillin 375 mg and metronidazole 250 mg, 3 times a day for 7 days	PPD ≥ 5 mm, BoP and/or SoP, as well as marginal bone loss ≥ 3 mm detected radiographically	Prophylaxis/oral hygiene instruction. Those patients who presented periodontitis were treated first	Local anesthesia, implant surfaces treated with ultrasonic devices with PEEK fiber tip and carbon fiber hand instruments//All patients rinsing with chlorhexidine 0.12%, two times a day for 4 weeks

**Table 2:** Details of patients’ characteristics and debridement intervention for the included studies.

Publication	Smoking Habits	History of Periodontitis	Implants Treated	Instruments Used for Debridement
Butcher 2004 [28]	Smokers 2/14 control group; 3/14 test group	Periodontal lesions treated; no report on history of periodontal disease	48 ITI SLA	Hand plastic instrument + chlorhexidine digluconate 0.2% solution
Park 2021a [16]	All patients were smoking < 10 cigarettes/day	No report on history of periodontal disease	78 implants, 1 per patient (39 in test group: 8 bone-level, external connection; 3 tissue-level, internal connection; 24 bone-level, internal connection, with micro-thread, 4 unknowns; 39 in control group: 10 bone-level, external connection; 7 tissue-level, internal connection; 21 bone-level, internal connection, with micro-thread, 1 unknown)	Oral hygiene products (FX2 brush, Complete Care toothpaste, 1 min interdental brush and Ultra floss) and non-surgical debridement with ultrasonic scaler + in test group minocycline hydrochloride dehydrate (10.0 mg) and metronidazole benzoate
Park 2021b [16]	All patients were smoking < 10 cigarettes/day	No report on history of periodontal disease	79 implants, 1 per patient (40 in test group: 8 bone-level, external connection; 6 tissue-level, internal connection; 26 bone-level, internal connection, with micro-thread; 39 in control group: 10 bone-level, external connection; 7 tissue-level, internal connection; 21 bone-level, internal connection, with micro-thread, 1 unknown)	Oral hygiene products (FX2 brush, Complete Care toothpaste, 1 min interdental brush and Ultra floss) and non-surgical debridement with ultrasonic scaler + in test group minocycline hydrochloride
Blanco 2022 [17]	Smokers 5/16 control group; 4/16 test group	History of periodontal disease 13/16 control; 9/16 test group	Mean implant included per patient 2.12 (range 1–5) control group; 1.75 (range 1–4) test group. No data for the implant type	Supra- and submucosal stainless-steel ultrasonic scaler + stainless steel curettes + chlorhexidine 0.12% irrigation
DeWaal 2021 [26]	Smokers 10/32 control group; 7/30 test group	18/32 in the control group and 15/30 in the test group had mild/moderate periodontitis (stage I or II); 14/32 in the control group and 15/30 in the test group had severe periodontitis (stage III or IV)	57 patients with 132 implants; in the control group: 29 patients, 64 implants (6 Alpha-Bio Tec, 1 Camlog, 2 Dentium, 20 Dentsply Sirona, 4 MIS, 16 Nobel Biocare, 14 Straumann, 5 Zimmer Biomet); in the test group: 28 patients, 68 implants (3 Camlog, 24 Dentsply Sirona, 3 Neobiotech, 24 Nobel Biocare, 13 Straumann, 7 Zimmer Biomet)	Air polisher with sub-gingival tip (EMS Air-flow^®^ with erythritol-based powder containing chlorhexidine (14 μm, Air-Flow ^®^ Powder PLUS, EMS)) + ultrasonic instruments only on exposed screw threads. In one to five sessions.
Shibli 2019 [25]	All 40 non-smokers patients	No report on history of periodontal disease	40 patients, 20 implants per group	Non-surgical peri-implant debridement performed with Teflon curettes in a single appointment
Polymeri 2022 [27]	Smokers 3/19 control group; 8/18 test group	History of periodontitis 7/19 in the control group; 9/18 in the test group	One implant per patient, in the control group 5 Nobel, 5 Straumann, 5 Biomet 3i, 4 other brands (*n* = 19); in the test group 9 Nobel, 4 Straumann, 1 Biomet 3i, 4 other brands (*n* = 18)	Ultrasonic devices with PEEK fiber tip and carbon fiber hand instruments

Two studies were judged at low [17,26], two at moderate [16,28], and two at high risk of bias [25,27] (Table 3).

### 2.1. Adjunctive Use of Local Antibiotics

The adjunctive use of local antibiotics was analyzed in two studies [16,28], with no baseline unbalance in the clinical conditions between the groups. None of the studies used a topical placebo as comparator.

An 8.5% doxycycline hyclate paste inserted in the peri-implant sulcus resulted statistically more efficacious in PPD, CAL and BOP improvement to non-surgical treatment over an 18-week study [28]. There were significant within-group changes in PPD, CAL and BOP between baseline and the 4-month follow-up (*p* < 0.001 in all comparisons).

At 12 weeks follow-up, the success rate (peri-implantitis resolution) was significantly higher using a prototype ointment based on minocycline hydrochloride dehydrate plus metronidazole benzoate (MM group; 12 cases) (*p* = 0.011) (Park 2021a [16]) or a commercial gel of minocycline hydrochloride (MC group; 8 cases) (Park 2021b [16]) than in the non-surgical treatment only (NST group; 1 case) (*p* = 0.011 and *p* = 0.040, respectively). Mean PPD and mean BOP improvements were significantly higher compared to NST in MM, but not in the MC group (1.95mm, ±1.28, *p* = 0.0023 and 0.51, ±0.32, *p* = 0.0381, respectively). Subgroup analyses were conducted for clinical changes at the deepest sites in the severe (PPD ≥ 8mm) and moderate group (5mm ≤ PPD <8mm). Statistically significant changes were found only in the group showing severe PPD, favoring the MM group (*p* = 0.024) compared to the NST and MC groups (*p* = 0.041). Data reported a 4.50 mm (±1.57) reduction from 9.08 mm (±1.16) in the MM group, 3.10 mm (±2.17) in the MC group from 9.10 mm (±1.14) and 2.33 mm (±1.59) from 9.06 (±0.77) in the MST group.

Considering PDD and BOP, the meta-analysis included the two studies [16,28] and showed improvements in favor of the use of local antibiotics for both the outcomes (PDD: mean 0.91; CI 0.16–1.68; BOP: mean 0.82; CI 0.15–1.50), considering standardized mean values.

Regarding the implant success rate (peri-implantitis resolution), the meta-analysis could include only the data from Park et al. 2021 [16]: at one year, the success rate was higher in test group than the control group (risk ratio 9.89; CI 2.39–40.89). Disease resolution was obtained in few cases, but it was significantly higher when local antibiotics were employed (20–30% vs. 2% of cases).

Butcher et al. 2004 declared the absence of implant failure [28], while this outcome was not clearly stated in the other study [16]. The other secondary outcome measures considered in this review were not reported.

### 2.2. Adjunctive Use of Systemic Antibiotics

Adjunctive systemic antibiotics were administered in four studies [17,25,26,27].

Systemic amoxicillin plus metronidazole was employed in three studies [25,26,27], three times a day following different protocols for one [26,27] or two weeks [25], as described in Table 2. Metronidazole alone was prescribed in one study for one week [17].

Follow-up visits were scheduled at 3 months [26,27] and 1 year [17,25].

Bone level changes were analyzed in two studies only [17,26]. Blanco et al. reported, at 1 year, a statistically significant bone gain of 2.33 mm in the test group vs. 1.13 mm in the control group (*p*-value < 0.05) [17], while no difference was found at 3 months by De Waal and colleagues [26]. The meta-analysis analyzed the 3-month values of the studies and no differences were found between groups.

Two studies showed PPD improvements favoring the test groups [17,25]. At one year, a PPD reduction of 3.1 mm (±1.2 mm) vs. 1.8 mm (±0.2 mm) (*p*-value < 0.05) was reported in the test group by Shibli 2019 [25], while a reduction of 2.53 mm (95% CI 1.37–3.69 mm) vs. 1.02 mm (95% CI 0.06–1.99mm) (*p*-value < 0.5) was found in the test group as reported by Blanco 2022 [17]. No differences were found in the two other studies [26,27]. The data at four months were considered for the-meta analysis and supported the use of adjunctive systemic antibiotics with a mean improvement of 1.15 mm (95% CI 0.31–1.99 mm).

PAL changes were reported in three studies [17,25,26]. At one year, Shibli et al. showed an improvement in the test group of 2.6 (±1.5) mm vs. 1.4 mm (±0.8) in the controls [25]. Similarly, Blanco et al. described a higher improvement in the test group compared to the control group, 2.14 mm (95% CI 0.97–3.30mm) versus 0.53mm (95% CI −0.33–1.39 mm), respectively [17]. The meta-analysis supported the use of systemic antibiotics, with a mean improvement of 1.10mm (95% CI 0.13, 2.08).

The one-year success rate (peri-implantitis resolution) was analyzed in all studies and no significant differences between groups could be found in the single studies nor in the meta-analysis. Overall, peri-implantitis resolution ranged from 2% to 65% of cases with systemic local antibiotics.

One study [26] reported no difference in the number of adverse events between the groups, while 11 patients in the control and 9 in the test group were scheduled for the surgical treatment; two implants were lost, but it was not specified in which group.

## 3. Discussion

The adjunctive use of local antibiotics in the non-surgical management of peri-implantitis improved the success rate at 1 year and was associated with a 3-month PPD and BOP reduction. The adjunctive use of systemic antibiotics resulted, instead, in improved PPD and PAL only. Disease resolution ranged from 2% to 65% of cases.

The correct prescription of antibiotics is a worldwide priority due to the increasing threat of super-infections, antibiotic resistance and side effects. The World Health Organization (WHO) has issued a global action plan (GAP) to address this problem [6], in which dentistry plays an important role since about 10% of global antibiotic prescriptions come from the dental profession [29]. The importance of using the antibiotics only when strictly necessary was highlighted in the 2019 policy statement of the FDI World Dental Federation [30]. Randomized controlled studies addressing the efficacy of both local and systemic antibiotics in preventing and treating oral infections is a priority [31]. Nonetheless, several alternative molecules are currently under investigation to find new agents showing antibacterial properties, thus contributing to reduce antibiotic resistance [32,33].

Local antibiotics have the advantages of achieving a high concentration in the target site, reducing risk of side effects and antibiotic resistance and being independent from patient compliance. The main disadvantages are the cost and the need for professional delivery. Butcher et al. reported the best result with adjunctive antibiotics, where the non-surgical therapy alone yielded small improvement in clinical parameters [28]. Non-surgical therapy alone gave greater improvements compared to baseline in Park et al. [16], where treatment was repeated at 3 and 8 weeks. In this study, the differences between test and control group were smaller, but the use of antibiotics gave greater improvement when they were applied in deeper pockets. The use of local antibiotics seems to be indicated when proper cleaning is more difficult, as in deep pockets, or could not be performed in optimal way. Adjunctive systemic antibiotics can be associated with antibiotic resistance, as described in some case reports [34]. The issue about the risk of peri-implant superinfection with opportunistic bacteria, viruses and yeast in immunocompetent patients has been put forward in a recent systematic review [35]. Antimicrobial testing has been suggested before systemic antibiotic usage [36], but it was not applied in the included studies.

The adjunctive application of local antibiotics appeared statistically superior to debridement alone in PPD reduction in several previous systematic reviews addressing non-surgical treatment of peri-implantitis [37,38,39]. This finding was confirmed in a Bayesian network meta-analysis [40]. Interestingly, these results were based on one study only [28], thus the reliability of this conclusion remains weak.

The results on the use of systemic antibiotics were based on four studies [17,25,26,27] that achieved opposite conclusions, but the meta-analysis showed statistical improvements in PPD and PAL. Interestingly, most of the difference favoring the use of antibiotics derived from studies in which non-surgical treatment alone resulted in a very low improvement from baseline [17,25], questioning the quality of the non-surgical therapy provided and the validity of the results of the meta-analysis. As for the local use of antibiotics, adjunctive antibiotics appeared more adequate when optimal biofilm removal could not be achieved. Moreover, every study used a different protocol for antibiotic administration, highlighting a lack of consensus in the treatment modality.

Recently, some systematic reviews focused on antibiotics applied locally [15,41], systemically [13] or in both modalities [12] to face the management of peri-implantitis, and their results were consistent with our systematic review. Passarelli et al. 2021 [15], in particular, addressed the use of local/topical antibiotics for both non-surgical and surgical treatment of peri-implantitis. Only two studies dealt with the non-surgical approach and they concluded that the administration of local/topical antibiotics could have a positive effect, but more studies were needed to confirm this assumption. Wang and co-workers 2022 further confirmed this finding [14]. In Javed et al. (2013) [12], the efficacy of antibiotics was questioned, while no significant effect of systemic antibiotics on PPD and BOP reduction was found by Toledano 2022 [13]. Non-randomized studies were included as well in both reviews. Moreover, the efficacy of alternative or adjunctive measures were investigated in another recent systematic review [42], which showed that systemic antibiotics provided better BOP reduction than a placebo, despite this assumption being based on only two studies.

The current systematic review is the first one using strict study selection and evaluation criteria and included the analysis of the most recent therapies available in the literature. Only RCTs on the adjunctive use of antibiotics, besides the mechanical debridement, were included, since mechanical biofilm disruption remains essential for antibiotics’ efficacy. Peri-implantitis should be defined according to the 2017 World Workshop on Periodontal Diseases, i.e., including cases with increased probing depth compared with previous examination, or PPD ≥6 mm and bone loss beyond the initial remodeling or ≥3 mm apical of the most coronal portion of the intraosseous part of the implant [1]. Four included studies [16,25,26,27] considered PPD > or ≥5m, but, since the mean value of the PPD was above 6 mm and the bone loss was reported, these studies were analyzed.

Considering that the periodontal treatment can promote a reduction in pocket probing depth of about 1–1.5 mm in pockets of 4–6 mm and of 2–2.5 mm in deeper sites exceeding 6 mm [43], a similar trend might be expected also for peri-implant lesions. Non-surgical therapy could induce a higher PPD reduction when applied on deep pockets, as shown in two included studies that analyzed the results in subgroups in relation to the baseline PPD [16,26]. Pockets deeper than 7 mm showed an improvement of 2.42 mm (±1.23) and 3.19 mm (±1.53) mm in the control group and in the test group, respectively, with no statistical difference between the two groups in the work by DeWaal et al. 2021. In Park 2021, the most severe group improved by 4.5 mm (±1.57) (MM group), 3.10 mm (±2.17) (MC group) and 2.33 mm (±1.59) (NST group) with significant differences between the groups. On the other hand, disease resolution, defined as PPD < 5 mm with no BOP, was obtained in few cases, but it was significantly higher when local antibiotics were employed (20–30% vs. 2% of cases) [16]. Disease resolution when using systemic antibiotics showed a great variability among the studies, ranging from 5% to 65% of cases, with no significant difference between groups considering the meta-analysis. This variability may be due to the different non-surgical protocol applied, to the different defects treated in the studies and the different types of implant affected by the disease. In a study on non-surgical therapy, chlorhexidine or placebo chips were re-inserted during the follow-up visits in presence of PPD deeper than 6 mm with promising results [44]. Re-treatment of the sites with inflammation was performed in Esposito et al. 2013 [45]: about 1.5 mm of PPD reduction was achieved (baseline PPD of about 6mm). Non-surgical re-treatment can be part of the maintenance therapy and the reduction of diseased sites can decrease the need for further invasive therapy. Interestingly, improvements were reported in the two studies with one-year follow up [17,25], suggesting the role of an adequate maintenance protocol. Supportive therapy plays a critical role in peri-implantitis treatment since, like periodontitis, it is a chronic disease that should be followed over time [46]. High patient- and implant-level survival rates were reported in the medium and long term when regular supportive care was provided [47].

The clinical significance of our results should consider the heterogeneity of the few studies included, which used different treatment protocols, enrolled a limited number of patients, included cases with different severity of the disease and mainly applied a short-term follow-up, at 4–6 months. The meta-analysis, in particular, could be performed considering very few studies, thus the resulting data must be interpreted cautiously. The most important outcome, i.e., implant loss as suggested by Graziani et al. [48], was rarely reported. Marginal bone level changes could provide reliable information on disease progression, but this outcome was used only in a few studies and it was analyzed at a short follow-up. In addition, only doxycycline, minocycline and metronidazole were examined, therefore, the conclusion could not be valid for other types of antibiotics, following different protocols for drug administration, highlighting a lack of consensus in the pharmacological management of this disease. Finally, only two studies were at low risk of bias, and studies at high risk of bias can overestimate the efficacy of an intervention. Multicenter randomized controlled studies are needed to further clarify the role of antibiotics in the treatment of peri-implantitis, in particular involving an adequate sample size, focusing on clinically important outcomes, such as implant failure and marginal bone level, and considering long-term follow-up. The protocol that would be applied, including the supporting maintenance therapy, should be described in detail together with the implant characteristics, such as the type of implant surface.

## 4. Materials and Methods

This review was written according to the PRISMA Statement for reporting Systematic Reviews and Meta-Analyses. The protocol was registered on PROSPERO (CDR42022326753).

The PICO format was prepared in association with the following research question: in patients with at least one dental implant affected by peri-implantitis, is the adjunctive use of antibiotics effective in the non-surgical treatment of peri-implantitis?

P: patients with at least one dental implant affected by peri-implantitis,

I: adjunctive use of systemic and/or local antibiotics,

C: non-surgical treatment of peri-implantitis,

O: implant failure, radiographic marginal bone loss, probing pocket depth (PPD) changes, bleeding on probing/pus changes, probing attachment level (PAL) changes, complications and side effects, peri-implantitis resolution (absence of PPD > 5mm, absence of BOP).

### 4.1. Inclusion and Exclusion Criteria

Only randomized controlled trials (RCTs) on humans, with parallel group and split-mouth design, evaluating interventions to treat peri-implantitis non-surgically, were included.

A clear definition of peri-implantitis cases should be explicitly stated and it should agree with the definition of peri-implantitis based on the 2017 World Workshop on Periodontal Diseases [1]:

- Presence of bleeding and/or suppuration on gentle probing.
- Increased probing depth compared to previous examinations.
- Presence of bone loss beyond crestal bone level changes resulting from initial bone remodeling.

In the absence of an available previous clinical examination, the diagnosis of peri-implantitis was based on the combination of:

- Presence of bleeding and/or suppuration on gentle probing.
- Probing depth of ≥6 mm.
- Bone level ≥ 3 mm apical to the most coronal portion of the intraosseous part of the implant.

A trial was excluded if no outcomes of interest were considered or were not clearly described; if data were given only at implant and not at patient level; or if data on mucositis and peri-implantitis were aggregated.

### 4.2. Outcomes

Primary outcome measures were:

- Implant success or failure (removal of previously osseointegrated implants because of mobility, progressive marginal bone loss or infection);
- Radiographic marginal bone level change on intraoral radiographs taken with a parallel technique;
- Probing pocket depth (PPD) change (mean and standard deviations, SD);
- Bleeding on probing/pus change (mean and SD);
- Complications and side effects: any reported complication or side effect;
- Probing ’attachment’ level (PAL) change (mean and SD);
- Peri-implantitis resolution: absence of PPD >5mm, absence of BOP.

Secondary outcome measures were:

- Recurrence of peri-implantitis;
- Marginal soft tissue recession;
- Patient-related outcomes (preference, aesthetic);
- Aesthetics evaluated by dentists;
- Cost (treatment time plus material costs).

### 4.3. Search Method for Identification of the Studies

Relevant articles were searched on electronic databases (MEDLINE via Pubmed; EMBASE via Ovid; EBSCO Dentistry and Oral Science Source). The last electronic search was performed on 4 August 2022.

The following combination of keywords (i.e., Medical Subject Headings MeSH) and free text terms was used: (Peri-implantitis) OR (periimplantitis) OR (peri-implant disease) OR (peri-implant infection) AND (clinical randomized trial) AND (non-surgical therapy) OR (non-surgical treatment) OR (nonsurgical treatment) AND (antibiotic*) OR (anti-infective agents) OR (Anti-bacterial agents) OR (Anti-biotic*) OR (anti-bacterial) OR (antibacterial) NOT (review) NOT (in vitro).

Hand searching was done on the following journals: Clinical Implant Dentistry and Related Research; Clinical Oral Implants Research; International Journal of Oral and Maxillofacial Implants; International Journal of Oral Implantology; Journal of Clinical Periodontology; Journal of Periodontology. The references of all selected full-text articles and of the reviews regarding non-surgical therapy or peri-implantitis were examined.

### 4.4. Study selection and Quality Assessment of Selected Articles

Two authors (MGG and MS) performed the first screening independently on titles and abstracts according to the inclusion criteria.

The full texts of all articles identified during the first screening were obtained and evaluated independently by two authors (MGG and MS) to check if the inclusion criteria were met. Disagreements about study inclusion and exclusion were resolved by discussion with a third author (MDF).

The Cochrane Collaboration‘s tool for assessing risk of bias (low, high, unclear) was used on all the selected articles. It included the following domains: random sequence generation, allocation concealment, blinding of participants and personnel, blinding of outcome assessment, incomplete outcome data, selective reporting and other bias [49]. Two authors (MGG, MS) performed the risk of bias assessment. Any doubt was resolved by discussion with a third author (MDF). The included studies were categorized at low risk of bias if all of the aforementioned domains obtained a low risk score, at moderate risk if one or more of the domains were scored as unclear and none as high risk, and at high risk if one or more domains were judged at high risk.

### 4.5. Data Extraction

A data extraction template was produced and filled (MGG, MS). It included study design, population (presence of periodontal disease, smoking status), case definition, observation period, pretreatment phase, maintenance, baseline mean values (and SD) and mean changes of the primary and secondary outcome variables, intervention, comparison and quality assessment domains. In case of missing information, the corresponding author of the article was contacted by e-mail to obtain complete data. If there was no reply, the same e-mail was sent to co-authors. If no answer was obtained, the study was excluded from the analysis. When feasible, missing standard deviations were estimated using the methods described in Section 7.7.3 of the Cochrane Handbook for Systematic Reviews of Interventions, Version 5.1.0.

### 4.6. Method of Analysis

Comparability of control and test group(s) at baseline was checked for the main outcomes, using unpaired Student’s t-test for continuous variables (e.g., PPD, CAL, bone loss) and Pearson’s chi square or Fisher’s exact test, as appropriate, for dichotomous data.

If there were at least two studies with similar interventions and comparable observation times, the data were combined by meta-analysis for each available outcome measurement. The statistical analysis unit was the patient, unless all compared studies expressed the results using the implant as the unit of analysis. Meta-analysis was performed for each outcome variable and heterogeneity was quantified through I^2^ statistics. Mean difference (MD) with a 95% confidence interval (CI) was used to synthesize the data for the continuous primary outcomes. Dichotomous data were analyzed using risk ratios (RR). Mean differences for continuous data were combined using fixed-effects models if no significant heterogeneity was detected (i2 < 60%, *p* > 0.05), otherwise (when i2 > 60% and *p* < 0.05) a random-effect model was adopted. The quantitative data were analyzed using Review Manager software (RevMan 5.4, Version 5.4.1 Copenhagen, Denmark: The Nordic Cochrane Centre, The Cochrane Collaboration, 2020).

## 5. Conclusions

Within the limitations of this systematic review, the use of adjunctive local antibiotics in the non-surgical treatment of peri-implantitis improved success rate, PPD and BOP reduction, while adjunctive systemic antibiotics improved PPD and PAL only. Diseased sites were reduced by the non-surgical treatments, but the success rate ranged a lot among different studies. The clinical significance of these results should consider that findings were based on only six studies; most of them were at uncertain or high risk of bias, showing different treatment protocols, involving few patients and applying a short-term follow-up. An adequate non-surgical debridement is pivotal, as well as the maintenance support therapy, since they correlate with the improvement of deep pockets, in particular if the treatment is repeated. Adjunctive antibiotics could be useful in those cases when the optimal mechanical debridement cannot be obtained.

## Figures and Tables

**Table 3:** Analysis of the risk of bias in the included studies.

Publication	Random Sequence Generation	Allocation Concealment	Blinding of Participants and Personnel	Blinding of Outcome Assessment	Incomplete Outcome Data Addresses	Selective Reporting	Other bias (Appropriate Statistical Analysis)	Overall Risk of Bias
Butcher 2004 [28]	Yes	Unclear	Unclear	Unclear	Yes	Unclear	Unclear	Moderate
Park 2021 [16]	Yes	Yes	Unclear	Unclear	Yes	Yes	Yes	Moderate
Polymeri 2022 [27]	Yes	No	Unclear	Unclear	Yes	Unclear	Yes	High
Blanco 2022 [17]	Yes	Yes	Yes	Yes	Yes	Yes	Yes	Low
De Waal 2021 [26]	Yes	Yes	Yes	Yes	Yes	Yes	Yes	Low
Shibli 2019 [25]	Yes	No	Unclear	Unclear	Unclear	Unclear	No	High

## Data Availability

Data are all in the manuscript.
